# Microbiome and Postbiotics in Skin Health

**DOI:** 10.3390/biomedicines13040791

**Published:** 2025-03-25

**Authors:** Santosh Kumar Prajapati, Lalitha Lekkala, Dhananjay Yadav, Shalini Jain, Hariom Yadav

**Affiliations:** 1 USF Center for Microbiome Research, Microbiomes Institute, University of South Florida Morsani College of Medicine, Tampa, FL 33612, USA; prajapati11@usf.edu (S.K.P.); lalithalekkala@usf.edu (L.L.); dhananjay11@usf.edu (D.Y.); jains10@usf.edu (S.J.); 2 Center of Excellence in Aging and Brain Repair, Department of Neurosurgery and Brain Repair, University of South Florida Morsani College of Medicine, Tampa, FL 33612, USA

**Keywords:** skin microbiome, skin health, postbiotics, skin diseases, acne, eczema, psoriasis, dermatology, skin barrier function

## Abstract

The skin microbiome, a diverse and dynamic ecosystem of microorganisms, plays a pivotal role in maintaining skin health by interacting with skin cells, immune components, and structural barriers. It is essential for skin homeostasis, immune defense, and protection against pathogenic colonization. Dysbiosis in the microbiome has been implicated in numerous dermatological conditions, including acne, eczema, psoriasis, and rosacea. Acne, the most prevalent skin condition, affects up to 85% of individuals at some point in their lives, while eczema and psoriasis impose significant public health and economic burdens. The composition of the skin microbiome varies across skin types and anatomical sites, with sebaceous, moist, and dry areas fostering distinct microbial communities. Emerging therapeutic strategies such as microbiome-targeted treatments offer novel avenues for addressing skin diseases. Among these approaches, postbiotics have gained significant attention for their safety and efficacy. Unlike probiotics, postbiotics are non-viable microbial cells or their metabolites, which reduce safety concerns while providing functional benefits such as UV protection and wound healing. This review consolidates current insights into the role of the skin microbiome in health and disease, emphasizing postbiotics as a promising therapeutic strategy by exploring the clinical and commercial potential of microbiome-based treatments, particularly postbiotics, and their ability to redefine dermatological care and improve patient outcomes.

## 1. Introduction

In recent years, the skin microbiome has garnered increasing attention in both scientific research and commercial skincare [1]. The skin is not only the body’s largest organ but also a dynamic ecosystem inhabited by trillions of microbes, including bacteria, fungi, and viruses, all of which contribute to skin health and functionality [2,3]. These microorganisms play a crucial role in maintaining the skin’s structural integrity and immune defense mechanisms by interacting with skin cells and environmental factors [4,5]. Hence, the microbiome is essential for the maintenance of skin barriers [5], and its dysbiosis has been implicated in a wide range of dermatological conditions, such as acne, eczema, and psoriasis [6]. Acne is the most prevalent skin condition, especially in adolescents, affecting up to 85% of people at some point in their lives [7]. Eczema, also known as atopic dermatitis, affects around 1–20% of the population, leading to chronic inflammation and skin barrier dysfunction [8]. Psoriasis, an autoimmune disorder, leads to scaly, itchy patches of skin, affecting about 3% of people worldwide [9]. Beyond these common conditions, rare disorders such as vitiligo (loss of skin pigmentation) and alopecia (hair loss) also impact millions of people [10].

The skin microbiome makes a significant contribution to cutaneous immune defense by preventing the colonization of pathogenic microorganisms and by supporting skin homeostasis [11,12]. However, when this delicate balance is disrupted, harmful bacteria can proliferate, worsening the symptoms—though not the root cause—of atopic dermatitis, rosacea, and seborrheic dermatitis [13]. For instance, *Staphylococcus epidermidis*, a commensal bacterium, produces antimicrobial peptides (AMPs) that suppress pathogens like *Staphylococcus aureus* while also modulating inflammatory pathways to support skin homeostasis [14]. Additionally, microbial metabolites, such as short-chain fatty acids (SCFAs) and ceramides, strengthen the skin barrier, promote hydration, and maintain an acidic pH, which is essential for preventing microbial overgrowth [15]. Further, commensal microbiome-specific T-cell responses, driven by skin-resident dendritic cells, occur without inflammation, highlighting tissue-resident cells’ ability to sense microbial changes [16]. Colonization by *Staphylococcus epidermidis* activates interleukin (IL)-17A+ CD8+ T cells, enhancing innate immunity and limiting pathogen invasion. Therefore, skin microbiomes have emerged as a critical therapeutic target in preventing and treating skin diseases.

The composition of the skin microbiome is affected by skin type (e.g., dry, oily, or moist), anatomical site, age, and environmental factors. Sebaceous sites, such as the forehead and back, are dominated by *Cutibacterium acnes*, a bacterium associated with sebum metabolism and acne pathogenesis [17]. Moist areas like the underarms harbor *Corynebacterium* and *Staphylococcus* species, while dry areas like the forearms have a higher microbial diversity, including Gram-positive and Gram-negative bacteria [18]. These variations reflect the adaptive nature of microbial communities to their local microenvironment. Overall, skin conditions, whether mild or severe, present significant challenges not only to individuals but also to public health systems globally. In the United States, the cost of managing skin diseases exceeds USD 75 billion annually, comprising direct costs like hospital visits, drugs, and over-the-counter treatments, as well as indirect costs such as loss of productivity and the psychological burden on patients [19]. Further, there are limited drugs available for the treatment of skin diseases, including anti-inflammatory agents, corticosteroids, and immunomodulators (tacrolimus, pimecrolimus, and methotrexate) [8]. Despite these advancements, available treatment options remain limited and often pose severe long-term adverse effects.

Recently, in clinical settings, therapies aimed at restoring microbial balance, such as the use of prebiotics, probiotics, and postbiotics, have shown promise in treating conditions like acne and atopic dermatitis [20,21,22]. Postbiotics represent a promising alternative for skin diseases due to their potential safety and efficacy profiles. Modulating this microbial community through microbiome-targeted approaches such as prebiotics, probiotics, and postbiotics is emerging as a promising therapeutic strategy [23]. Among these strategies, postbiotics, i.e., bioactive compounds produced by probiotic organisms, are gaining interest for their potential to support and restore skin microbial balance, offering unique advantages over prebiotics and probiotics [20,21]. Unlike probiotics, postbiotics lack live cells, which minimize safety concerns related to microbial viability, yet they retain beneficial properties that promote a healthy skin environment. Unlike prebiotics, which serve as nutrients for beneficial skin bacteria [24], postbiotics deliver immediate functional benefits without the need for live bacteria. Postbiotics, comprising non-viable microbial cells or their metabolites, have demonstrated promise in enhancing skin health by providing anti-inflammatory and antioxidant effects, strengthening the skin barrier, boosting hydration, and selectively supporting the growth of beneficial microbes while inhibiting pathogenic colonization [25,26]. This review aims to emphasize the skin microbiome and associated abnormalities. Further, we elaborated the findings on microbiome-targeted therapies in dermatology, with a primary focus on postbiotics’ role in maintaining microbial balance and supporting skin health.

## 2. Ontology of Skin

The skin is a complex, multilayered organ composed of epidermis, dermis, and hypodermis. These layers perform vital functions that contribute to the skin’s role as a physical barrier, thermoregulator, and sensory organ [27]. The epidermis, the outermost layer, is primarily made up of keratinocytes, cells that produce keratin, a structural protein crucial for the skin’s protective and resilient qualities [28,29]. The dermis beneath it contains connective tissue, blood vessels, nerves, and skin appendages such as hair follicles and sweat glands. The deepest layer, the hypodermis, consists of adipose tissue, which provides insulation and mechanical protecting to the body [30]. The skin microbiome is distributed throughout different layers and regions of the skin, with specific types of microorganisms adapted to different skin environments.

**Epidermis**: The outermost layer of the skin, particularly the stratum corneum (the top layer of the epidermis), hosts a variety of bacteria, fungi, and viruses [31,32]. This layer provides a protective barrier, and its slightly acidic pH favors the growth of certain beneficial bacteria that help defend against pathogens [32]. The diverse community of microorganisms residing on the epidermis primarily consists of bacteria, fungi, and viruses. Key species include *Staphylococcus epidermidis*, *Propionibacterium*, *Corynebacterium*, *Micrococcus*, and *Malassezia*, which predominantly inhabit the outermost layer of the epidermis, known as the stratum corneum [3]. Colonization by *Staphylococcus epidermidis* triggers the activation of IL-17A+ CD8+ T cells, which migrate to the epidermis, boost innate barrier immunity, and reduce pathogen invasion [16]. The T-cell responses specific to commensals arise from the coordinated activity of skin-resident dendritic cell subsets and occur without causing inflammation [16]. *Micrococcus* species are part of the normal flora of human skin and are involved in the defense against pathogenic microorganisms [33]. They produce antimicrobial substances and help maintain skin integrity by regulating the immune system. This indicates that tissue-resident cells are primed to detect and react to changes in microbial communities.

**Hair follicles (HF)**: Microorganisms also inhibit hair follicles, which provide a protected, nutrient-rich environment. Immune cells, including Langerhans cells (LCs), are present in the skin and around appendages, closely interacting with the microbiota [34]. LCs, located in the epidermis and HF outer root sheath, capture microbial antigens and interact with skin-resident memory T cells [35]. Under steady conditions, LCs promote the activation of T regulatory cells for peripheral tolerance. During pathogen exposure, they stimulate effector memory T cells. Additionally, microbe-derived products are taken up by antigen-presenting cells, keratinocytes, or diffuse through the epithelial barrier [36]. Skin-resident CD103+ dendritic cells sense microbial shifts, particularly in response to *Staphylococcus epidermidis* colonization in mice [37]. Further, in anaerobic conditions, the deeper follicle regions support the growth of bacteria like *Propionibacterium acnes*, commonly associated with acne [12,38]. Moreover, bacteriocins are antimicrobial peptides produced by bacteria to inhibit the growth of closely related or competing bacterial species and genera. *Staphylococcus epidermidis*, a commensal bacterium found on human skin, produces several types of bacteriocins that specifically target and inhibit pathogens, including *Staphylococcus aureus*, which is associated with infections and inflammatory skin diseases [39]. *Staphylococcus epidermidis* also produces antimicrobial peptides (AMPs) like phenol-soluble modulins (PSMs), which have strong antimicrobial activity against *Staphylococcus aureus* and other pathogens [40]. These AMPs help protect against skin infections and contribute to immune modulation by activating host immune receptors. For instance, PSMs can stimulate keratinocytes to release cytokines that enhance skin immunity and reinforce the skin’s barrier function [14].

**Sebaceous (oil) glands**: These glands produce sebum, an oily substance that supports certain types of bacteria and fungi, including *Cutibacterium acne*. Sebaceous areas, such as the scalp, face, and upper back, tend to have higher microbial density due to the abundance of lipids. As people age, sebum production gradually declines, especially after middle age [17]. This reduction in oiliness leads to less favorable conditions for *Cutibacterium acnes*, which explains why acnes often become less severe or even resolves as people grow older [17]. Additionally, the skin becomes dry with age, reducing the presence of lipid-dependent bacteria in hair follicles. Studies have consistently shown that the microbial communities in these distinct microenvironments differ significantly in both composition and function [41]. These variations are likely influenced by differences in the availability and utilization of nutrients by microbes, as well as the inhibitory effects of skin secretions unique to each site [42,43].

**Sweat glands**: Both eccrine and apocrine sweat glands contribute to the skin microbiome. Apocrine glands, particularly in areas like the armpits and groin, release a nutrient-rich sweat that supports different microbial communities, which are involved in body odor production [31]. However, the skin barrier is not solely dependent on its structural components. The resident skin microbiome, an ecosystem of bacteria, fungi, viruses, and archaea, plays a vital role in maintaining skin homeostasis and health [31]. The microbiome supports the skin by producing AMPs, regulating pH, and metabolizing sebum, the skin’s natural oil [5]. Importantly, this symbiotic relationship between skin cells and microbiota enhances the skin’s ability to protect against environmental insults, pathogens, and inflammatory stimuli [3]. The following section emphasized the skin microbiome and its interaction with different skin cells.

## 3. Commensal Microbiome and Immune Cell Interaction to Maintain Skin Health

**Figure 1:**
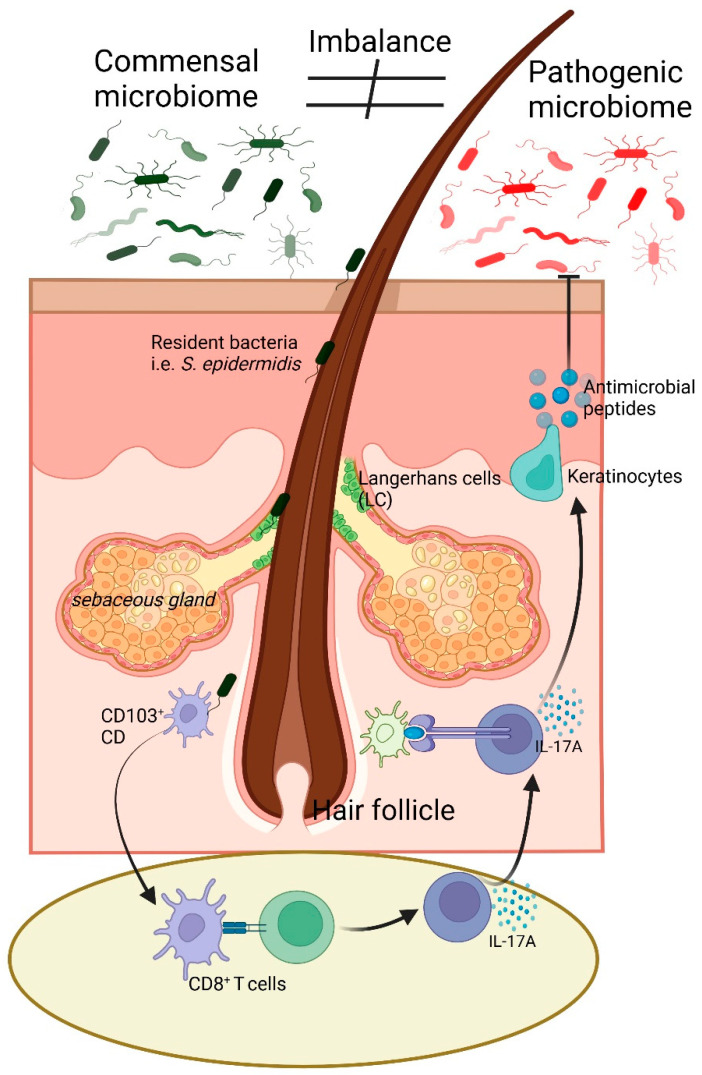
Illustration of skin microbiome balance and immune response regulation. The commensal microbiome, including *Staphylococcus epidermidis*, supports skin homeostasis and immune balance. An imbalance can lead to a shift towards a pathogenic microbiome, triggering immune responses. LC and CD103+ dendritic cells detect microbial changes and interact with CD8+ T cells, leading to IL-17A secretion. Keratinocytes respond by producing antimicrobial peptides, contributing to pathogen defense. The sebaceous gland and hair follicle microenvironment play key roles in this process. Arrow (→) indicates direction of flow of signals, while block arrow (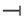) indicates inhibitory pathway.

The human skin is the body’s largest organ and serves as a complex and dynamic barrier that interacts with both the external environment and internal physiological systems [3]. In the skin resides a diverse community of microorganisms, collectively known as the skin microbiome, which includes bacteria, fungi, viruses, and archaea [31]. These microorganisms are essential for maintaining the health and function of the skin. They contribute to various processes, such as protecting the skin from pathogens, modulating immune responses, and maintaining the skin’s barrier function (Figure 1) [5].

The interaction between the skin and its resident microbiota is a critical determinant of skin health, and disruptions to this relationship can lead to a wide range of skin disorders.

The skin microbiome varies significantly across different body sites, largely influenced by factors such as moisture levels, temperature, pH, and exposure to environmental elements [3]. Areas such as the face, hands, and scalp have distinct microbial communities and are regulated by specific environments [3]. Understanding the complex interactions between skin cells and the microbiome is essential for developing effective treatments for skin diseases and for maintaining overall skin health.

## 4. Interaction of Microbiome with Skin

The skin microbiome varies across different regions of the body due to differences in environmental factors such as moisture, temperature, and sebum production [3]. Sebaceous areas like the forehead and upper back are rich in lipophilic bacteria such as *Cutibacterium acnes* (formerly *Propionibacterium acnes*), which metabolizes sebum into fatty acids that nourish the microbiome while maintaining skin integrity [44]. Conversely, moist areas like the axillae and groin support the growth of bacteria such as *Staphylococcus* and *Corynebacterium* species, which are better adapted to these humid microenvironments [18]. Skin microbiota can modulate the skin’s immune system by engaging with immune cells such as Langerhans cells and dermal dendritic cells [45,46]. These interactions help maintain immune tolerance, preventing overactivation of inflammatory pathways that may contribute to conditions such as atopic dermatitis and psoriasis [16]. Additionally, the skin microbiome produces a variety of metabolites, such as SCFAs, bacteriocins, and AMPs, which contribute to pathogen inhibition and skin barrier reinforcement. These metabolites play a significant role in maintaining skin homeostasis, promoting immune tolerance, and defending against potential pathogens [47,48]. For instance, *Staphylococcus epidermidis*, a commensal bacterium, has been shown to produce AMPs that directly inhibit *Staphylococcus aureus*, a pathogen associated with various skin infections [14]. Further, SCFAs such as acetate, propionate, and butyrate are key microbial metabolites produced primarily by anaerobic bacteria like *Cutibacterium acne*. These SCFAs help maintain an acidic environment on the skin, which is less hospitable to many pathogenic bacteria. SCFAs also strengthen the skin’s barrier function by promoting ceramide production, which enhances hydration and structural integrity [15]. Overall, these microbial metabolites not only inhibit pathogens but also strengthen the skin barrier. For example, commensal bacteria induce the production of tight junction proteins in skin cells, which improves barrier integrity and reduces transepidermal water loss. This activity is particularly beneficial in conditions where the skin barrier is compromised, such as eczema and psoriasis [15].

## 5. Types of Skin and Their Microbiomes

**Rough vs. smooth skin:** Rough and smooth skin are differentiated by the structure and composition of the stratum corneum, the outermost layer of the epidermis. Rough skin typically has a thicker stratum corneum, characterized by an accumulation of dead skin cells and lower hydration levels, which results in a coarse texture. In contrast, smooth skin is well-hydrated, with a balanced turnover of skin cells, leading to a soft and even texture [2]. The microbiome of rough skin tends to differ from that of smooth skin due to variations in the skin’s microenvironment. Rough skin, often found in areas subjected to friction or environmental stress, may harbor a higher concentration of Gram-positive bacteria such as *Corynebacterium* and *Staphylococcus* [49]. A study demonstrated that these bacteria thrive in dry, keratinized environments. They have adapted to metabolize lipids and proteins from the outer skin layer, contributing to the removal and renewal process of dead skin cells [43]. *Corynebacterium* species is known for their ability to degrade long-chain fatty acids in the skin [50], a process that can lead to odor production in rough areas like armpits but also supports the removal of dead cells in dry regions. Further, rough skin areas tend to host more bacteria, which can exacerbate inflammation. For instance, an increase in the diversity of certain *Staphylococcus* species, like *Staphylococcus epidermidis*, has been correlated with mild irritation in rougher areas of the skin [43]. These bacteria, while part of the normal skin flora, may become problematic when present in excessive amounts, leading to minor infections or increased cell turnover. Therefore, the microbial diversity of rough skin could be one of the contributing factors for the development of skin sensitivity or related abnormality. Application of microbiome/their metabolites or postbiotics may reverse these changes.

In contrast to rough skin, smooth skin tends to have a more diverse microbial community, including both Gram-positive and Gram-negative bacteria, fungi, and other microbes [3]. The presence of species like *Cutibacterium acne* and *Malassezia* (yeast) is often observed in more balanced skin areas [3]. This microbial diversity supports skin homeostasis, reducing the likelihood of inflammatory or pathogenic bacterial overgrowth. The diversity of microbes on smooth skin plays a key role in maintaining the integrity of the skin barrier. A study by Belkaid and Segre demonstrated that microbial diversity enhances the skin’s defense mechanisms by preventing pathogenic organisms from dominating the skin’s ecosystem [51]. Species like *Staphylococcus epidermidis*, in smoother skin areas, have been shown to produce antimicrobial peptides that help fend off pathogens like *Staphylococcus aureus*, thus contributing to the maintenance of skin health [51].

**Dark vs. light skin:** The color of human skin is determined by the amount and distribution of melanin, a pigment produced by melanocytes in the epidermis [52,53]. Individuals with darker skin have more melanin and often more active melanocytes compared to those with lighter skin [52,53]. This difference in pigmentation has implications for the skin’s interaction with UV radiation, with darker skin providing more protection against sun damage [54]. The skin microbiome also varies between individuals with dark and light skin. Studies have shown that individuals with darker skin tend to have a higher abundance of *Corynebacterium* species [18], while those with lighter skin may harbor more *Propionibacterium* species [44]. These differences may be attributed to variations in sebum production, moisture levels, and other factors influenced by melanin content. Melanin itself has been shown to possess antimicrobial properties, which could also influence the composition of the skin microbiome. Therefore, regulation or transplantation of a specific microbiome can regulate the skin melanin content and could be rate limiting in a microbiome-based therapeutic approach related to melanin sensitivity.

**Oily vs. dry skin:** Oily skin is characterized by the over-production of sebum, while dry skin has insufficient sebum production, leading to a lack of moisture and a compromised skin barrier [55]. The microbiomes of oily and dry skin differ significantly due to these variations in the skin’s lipid content. It has been reported that oily skin tends to have higher levels of *Cutibacterium acnes* (formerly *Propionibacterium acnes*), a bacterium that metabolizes sebum into free fatty acids [56]. These fatty acids can have antimicrobial effects, shaping the composition of the microbiome. Additionally, the abundance of sebum on oily skin can create a favorable environment for lipophilic fungi such as *Malassezia* [57]. In contrast, dry skin is more prone to colonization by bacteria like *Staphylococcus epidermidis*, which can thrive in lower-lipid environments [58]. The reduced microbial diversity on dry skin can make it more susceptible to irritation and inflammation.

**Sweaty vs. less sweaty skin:** Sweaty skin is typically found in areas with higher densities of sweat glands, such as the armpits, palms, and feet. Sweat, composed of water, salts, and organic compounds, creates a moist environment that influences the composition of the skin microbiome [59]. Moist environments promote the growth of bacteria like *Corynebacterium* and *Staphylococcus*, which are commonly found in sweat-prone areas. Less sweaty skin, on the other hand, tends to have lower moisture levels and a different microbial composition, and it has been demonstrated that these areas may harbor more *Actinobacteria* and *Firmicutes*, which thrive in drier conditions [60]. Therefore, differences in microbial composition between sweaty and less sweaty skin can influence the skin’s overall function and susceptibility to infections, as sweat can also act as a medium for the spread of pathogenic microorganisms if not regulated properly by the microbiome.

**Male vs. female skin:** The skin of males and females exhibits physiological differences, largely influenced by hormonal factors. Male skin tends to be thicker and produces more sebum due to the higher levels of androgens, such as testosterone [61]. This increased sebum production creates a favorable environment for sebaceous gland-associated bacteria like *Cutibacterium acnes* [62]. Additionally, the higher density of sweat glands in males contributes to a microbiome dominated by moisture-loving bacteria like *Corynebacterium* [63]. Female skin, in contrast, is typically thinner and may be more sensitive to hormonal fluctuations, particularly those related to the menstrual cycle [58]. These hormonal changes can influence both sebum production and the composition of the skin microbiome [58]. Females may experience shifts in their skin’s microbial community over time, with increases in *Staphylococcus* species during periods of lower sebum production [64]. The interplay between hormones, sebum, and the microbiome in males and females creates distinct microbial environments that influence skin health and susceptibility to conditions like acne and eczema.

## 6. Microbiome and Skin Diseases

**Figure 2:**
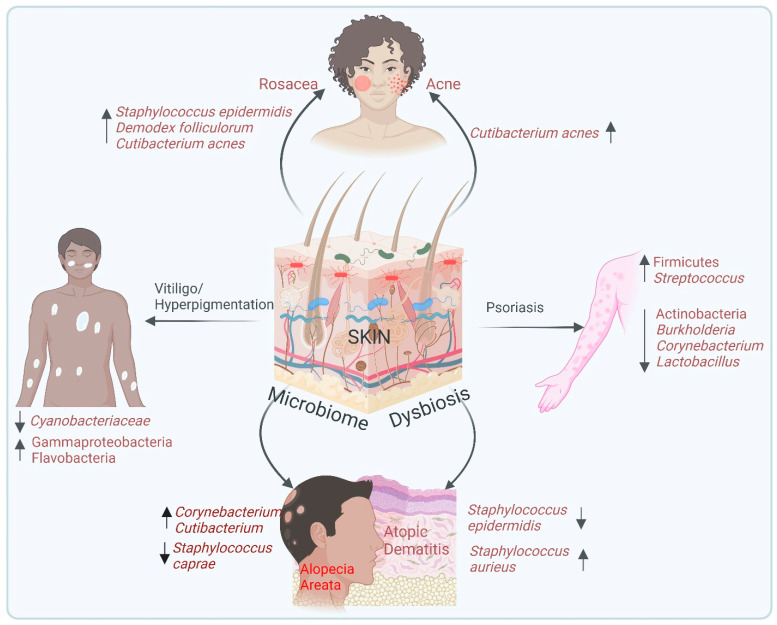
Skin microbiome dysbiosis in different dermatological abnormalities. This illustration highlights the association between skin microbiome dysbiosis and various dermatological conditions. Acne occurs due to an increase in the abundance of *Cutibacterium acne*. Rosacea causes increased levels of *Staphylococcus epidermidis*, *Demodex folliculorum*, and *Cutibacterium acne*. Further, during psoriasis, there is an enrichment of Firmicutes and *Streptococcus* and a reduction in actinobacteria, *Burkholderia*, *Corynebacterium* and *Lactobacillus*. Vitiligo/hyperpigmentation causes a reduction in the diversity of *Cyanobacteriaceae* and Flavobacteria. Atopic dermatitis is due to the dysregulation of the microbiome with increased *Staphylococcus aureus* and decreased *Staphylococcus epidermidis*. Moreover, the alopecia areata expresses high levels of *Corynebacterium* and *Cutibacterium* with a reduction in *Staphylococcus caprae*. This figure underscores the significance of microbial balance in maintaining skin health and that of its perturbation as a contributing factor to disease pathogenesis.

**Dysbiosis and skin disease:** Human skin, though resilient, is susceptible to a wide array of diseases that range from mild, cosmetic concerns to more severe conditions that significantly impair quality of life [65]. Skin disorders are among the most common health complaints globally, with billions spent annually on treatments and consultations [66]. Disruptions in the composition of the skin microbiome, known as dysbiosis in skin, have been linked to several inflammatory diseases, including acne, eczema and psoriasis [58], as well as other rare disorders like vitiligo, melanoma, and alopecia. In this context, various studies have shown that manipulating the microbiome can regulate skin health. For example, germ-free mouse models, which lack a commensal microbiome, exhibit heightened susceptibility to inflammatory skin conditions, but colonization with specific commensal bacteria such as *S. epidermidis* in these models restores immune balance and enhances skin integrity [37]. Despite this advancement, many skin diseases remain inadequately treated due to our limited understanding of their underlying pathophysiology and the ineffectiveness or adverse side effects of current treatments. Further, understanding how the skin microbiome contributes to skin diseases opens up new avenues for therapeutic interventions that target microbial balance rather than just addressing symptoms. The following sections provide an in-depth look at the relationship between the microbiome and specific skin diseases, as shown in Figure 2. Further, the skin microbiome plays a vital role in wound healing by maintaining a balance between pathogenic and non-pathogenic microbes [67,68]. While beneficial microbes such as *Staphylococcus epidermidis* and *Cutibacterium acnes* support immune modulation and prevent biofilm formation, pathogenic bacteria like *Staphylococcus aureus* and *Pseudomonas aeruginosa* contribute to inflammation and tissue damage [67,68]. Wound colonization is influenced by factors such as immune status, genetic predisposition, environment, and lifestyle [69]. Acute wounds, including those from trauma or burns, typically host a diverse microbial population comprising Gram-positive and Gram-negative bacteria [70]; whereas chronic wounds, such as diabetic foot ulcers, often harbor a stable pathogenic community dominated by *Staphylococcus aureus*, *Pseudomonas aeruginosa*, *Corynebacterium*, and anaerobes [67,71]. The presence of biofilms in chronic wounds further complicates healing by promoting inflammation and delaying tissue regeneration [67]. Therefore, understanding the wound microbiome is crucial for developing targeted therapeutic strategies to enhance healing and prevent complications.

**Acne:** Acne is characterized by the formation of pimples, blackheads, whiteheads, and cysts, primarily on the face, chest, and back [72]. *Cutibacterium acnes* (formerly *Propionibacterium acnes*) is the primary bacterium implicated in acne development [56,73]. This microorganism increases in the oily environment of sebaceous glands and induces inflammation by interacting with Toll-like receptors (TLRs) on keratinocytes, leading to the release of pro-inflammatory cytokines such as IL-6, IL-1β, and TNF-α [74]. Additionally, *Cutibacterium acnes* contribute to inflammation and host tissue damage by producing various virulence factors, including lipases, proteases, and Christine–Atkins–Munch-Petersen (CAMP) factors [75]. A study revealed that acne-associated *Cutibacterium acnes* strains significantly increased the production of inflammatory cytokines—specifically interferon (IFN)-γ and IL-17—in peripheral blood mononuclear cells [76]. Another study by Agak et al. emphasized the increased expression of IFN-γ and IL-17 in acne lesions [77]. Overall, an elevated level of *Cutibacterium acnes* enhanced the expression of filaggrin and integrin, contributing to the abnormal adhesion and differentiation of keratinocytes [78]. Moreover, acne-associated *Cutibacterium acnes* strains exhibited significantly higher expression of an adhesion protein, a cell surface hydrolase, compared to strains linked to healthy skin. This indicates the distinct functional roles of *Cutibacterium acnes* strains in modulating host immune responses and acne pathogenesis. These findings highlight the critical role of the microbiome in regulating acne. Therefore, a balanced skin microbiome is essential to prevent the overgrowth of pathogenic strains, suggesting that microbial modulation may be a promising approach to managing acne.

**Eczema (Atopic Dermatitis):** Eczema is a chronic inflammatory skin condition characterized by red, itchy, and inflamed skin [79]. The disease often manifests in childhood and can persist into adulthood, significantly impacting quality of life. The exact cause of eczema is multifactorial, involving genetic predisposition, environmental triggers, and immune dysregulation [79]. In recent years, the role of the skin microbiome in eczema has garnered significant attention. One of the hallmark features of eczema is a reduction in microbial diversity, particularly a decrease in beneficial bacteria such as *Staphylococcus epidermidis* and an overgrowth of *Staphylococcus aureus* [80]. *Staphylococcus aureus* can exacerbate the inflammatory response in eczema by colonizing the skin and producing toxins that further damage the skin barrier [81]. Studies have shown that the presence of *Staphylococcus aureus* on the skin correlates with increased disease severity as the bacterium promotes immune activation and the release of pro-inflammatory cytokines like IL-4, IL-13, and IL-31 [82]. *Staphylococcus aureus* can aggravate the severity of eczema by releasing various virulence factors such as superantigens (SAgs). Mechanistically, SAgs bind to the major histocompatibility complex class II (MHC-II) molecules on antigen-presenting cells (APCs), including skin keratinocytes, and to the β-chains of T cell receptors (TCRs) [83]. This results in the non-specific activation of T cells, triggering systemic inflammation through the release of pro-inflammatory cytokines such as TNF-α and IL-1β [84]. Overall, in eczema, reduced microbial diversity and the overgrowth of *Staphylococcus aureus* exacerbate the disease by intensifying inflammation and disrupting the skin barrier. Therefore, findings suggest that the regulation of *Staphylococcus aureus* could present a viable therapeutic approach to treating or preventing eczema.

**Psoriasis:** Psoriasis is a chronic autoimmune condition that affects approximately 2–3% of the global population. It is characterized by thick, scaly patches of skin that can appear on different parts of the body, which includes the scalp, knees, and lower back [85]. Psoriatic skin lesions exhibit an enrichment of bacteria from phyla of Firmicutes, whereas bacteria from phyla of actinobacteria are notably under-represented compared to healthy and non-lesional skin [86].

Furthermore, *Streptococcus*, *Staphylococcus*, Corynebacterium, and Propionibacterium were identified in lesional skin [87]. Compared to healthy individuals, those with psoriasis often have lower microbial diversity and an enrichment of *Streptococcus* and reduction in *Burkholderia*, *Corynebacterium*, and *Lactobacillus* species [2,88,89]. In particular, *Streptococcus pyogenes* has been implicated in triggering and exacerbating psoriasis by inducing immune responses that promote the proliferation of keratinocytes and inflammatory cells [90,91]. Additionally, Chang et al. showed that a reduction in immunoregulatory bacteria like *Staphylococcus epidermidis* and *Cutibacterium acnes* leads to increased colonization of *Staphylococcus aureus*, which aggravates cutaneous inflammation through the T helper type-17 (Th17) axis [92]. This condition is driven by the hyperactivation of the immune system that leads to the rapid turnover of skin cells. Although psoriasis has a strong genetic component, environmental factors, including microbial dysbiosis, play a critical role in its pathogenesis [93]. Research on the microbiome in psoriasis has revealed significant alterations in the skin’s microbial communities.

**Rosacea:** Rosacea is a persistent inflammatory skin condition that mainly impacts the face, leading to symptoms such as redness, visible blood vessels, and, in some instances, small, red, pus-filled bumps [94]. While the precise cause of rosacea remains unclear, it is believed to result from a combination of genetic factors, immune system dysfunction, and environmental triggers. Recently, skin microbiomes have emerged as a key player in rosacea pathogenesis. Individuals with rosacea often show dysbiosis in their skin microbiomes, particularly an overgrowth of the skin mite *Demodex folliculorum*, which can provoke an inflammatory response [95]. Additionally, studies have found that *Cutibacterium acnes* and *Staphylococcus epidermidis* are more abundant on the skin of rosacea patients, potentially contributing to the condition’s characteristic inflammation [96,97]. The interactions between these microorganisms and the immune system can exacerbate rosacea symptoms, leading to the release of pro-inflammatory cytokines and activation of immune cells to the affected areas. Targeting microbiome through therapies that reduce dysbiosis offer new treatment opportunities for individuals with rosacea.

**Sunburn:** Sunburn is caused by overexposure to ultraviolet (UV) radiation, leading to damage to the skin’s outer layers, inflammation, and pain [98]. While sunburn is an acute injury, repeated or severe sunburns can increase the risk of skin cancer and premature skin aging [99]. UV radiation not only affects skin cells but also alters the composition of the skin microbiome [100]. UV radiation can reduce the diversity of the skin microbiome, making the skin more vulnerable to harmful bacteria that can exacerbate inflammation and hinder the healing process. In healthy skin, the microbiome plays a protective role by modulating immune responses and promoting tissue repair [100]. Microbiome-based treatments, such as postbiotic formulations, are being explored as potential strategies to promote faster recovery from sunburn by enhancing the skin’s microbial diversity and reducing inflammation [101]. These treatments aim to restore the skin microbiome’s protective function, mitigating UV-induced damage and accelerating healing. Recently, Ácsová et al. demonstrated that *Lactococcus Ferment Lysate* and *Bifida Ferment Lysate* inhibit the synthetic DPPH (2,2-diphenyl-1-picrylhydrazyl) radical and have a sun-protective capbility with SPF (sun protective factor) 4-5 against UVB radiation [102]. Similarly, Kimoto-Nira et al. demonstrated that oral intake of heat-killed cells of the *Lactococcus lactis* strain H61 reduced the dehydration of the skin and enhanced skin elasticity [103]. Moreover, fermented milk from the *Lactobacillus helveticus* strain showed therapeutic potential for UVB-exposed skin by reducing lipid peroxidation and melanin formation [104]. Therefore, postbiotics-based skin formulation could be a promising strategy for sunburn protection and some age-related skin property alterations, such as changes to the melanin content.

**Vitiligo and hyperpigmentation:** Vitiligo is a persistent skin condition characterized by the loss of pigmentation due to damage of melanocytes [105]. This causes white patches to develop on various parts of the body. Vitiligo is recognized as an autoimmune disorder in which the immune system erroneously attacks melanocytes, though the exact mechanisms remain unclear [105]. Topical corticosteroids (TCSs) and topical calcineurin inhibitors (TCIs) are mainly used for the treatment of many types of vitiligo [106]. However, long-term use of these treatments has serious side effects; therefore, there is a need for alternative treatment strategies. Hyperpigmentation is a condition characterized by darkened patches of skin caused by an overproduction of melanin, often following inflammation or injury [107]. Emerging research has indicated a potential role in the skin microbiome in vitiligo’s pathogenesis. Studies have shown that individuals with vitiligo often exhibit dysbiosis, or microbial imbalance, particularly on depigmented areas of the skin [108]. This microbial imbalance may influence immune responses, further exacerbating melanocyte destruction and accelerating depigmentation [108]. In vitiligo patients, research indicates a decrease in the diversity of skin microbes, with studies showing a reduction in beneficial bacteria like *Corynebacteriaceae*, while potentially seeing an increase in bacteria like Gammaproteobacteria and Flavobacteria, suggesting an imbalance in the skin microbiome within affected vitiligo patches; essentially, the microbial diversity on vitiligo-affected skin tends to decrease compared to that of healthy skin. Therefore, alterations in skin bacteria have been observed to correlate with inflammation, suggesting that microbial shifts could act as additional triggers for autoimmunity in vitiligo patients [109].

**Alopecia areata (AA):** Alopecia areata causes abrupt patches of hair loss, usually on the scalp. The disorder results in the loss of hair follicles. Numerous studies have demonstrated the significant potential of using platelet-rich plasma (PRP) against AA [110]. However, due to its low stability and the lack of standardization of the production process, topical applications are difficult. This limitation has been resolved by Rinaldi et al., who used bioactive peptides obtained from biotechnological tools and combined them with postbiotics [111]. Numerous investigations into the scalp microbiomes of AA patients have yielded conflicting results. For instance, Won et al. investigated severe instances of AA and observed a decline in *Staphylococcus caprae* from the Firmicutes phylum and a notable rise in bacteria from the Actinobacteria group, including *Corynebacterium* and *Cutibacterium* [112]. On the other hand, Juhasz et al. found that individuals with AA had more Clostridia, a different class of Firmicutes, on their scalps [113]. Furthermore, studies have demonstrated that patients with AA have higher levels of *Neisseria* and *Anaerococcus* in their epidermis, while *Candidatus aquiluna* and *Staphylococcus epidermidis* are lower in their dermis [114,115]. Thus, these findings are providing a window for research into AA in context to microbiome regulation for the purpose of developing a better treatment strategy.

Overall, based on the evidence, we can conclude that disruption of microbial diversity is one of the key factors underpinning the regulation of skin-related abnormalities. Therefore, microbiome modulators could be a better strategy to employ. Thus, in the following section, we elaborate the microbiome modulator in the treatment of skin diseases.

## 7. Postbiotics in the Treatment of Skin Diseases

**Figure 3:**
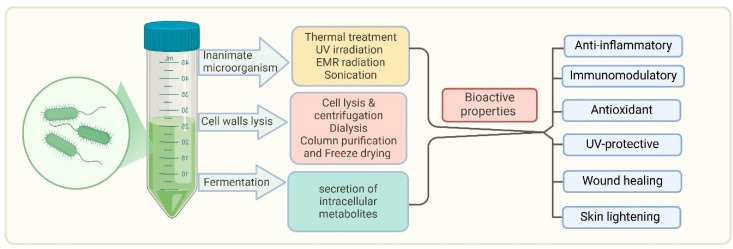
Production and bioactive properties of postbiotics. This diagram illustrates the process of obtaining bioactive properties from microorganisms through various treatments such as thermal treatment, UV irradiation, EMR radiation, sonication, cell wall lysis, and fermentation. Fermentation leads to the secretion of intracellular metabolites that contribute to postbiotic activity. Bioactive properties include diverse therapeutic effects against acne, rosacea, psoriasis, atopic dermatitis, and vitiligo/hyperpigmentation. It also shows immunomodulatory, UV-protective, and wound healing properties. This highlights the promising therapeutic potential of postbiotics in maintaining health and addressing skin-related issues.

Postbiotics refer to bioactive compounds. These components can modulate immune responses, enhance skin barrier function, and reduce inflammation, making them valuable tools in skincare and dermatological treatments. Viable cells can be deactivated using various mechanical approaches, such as sonication, heat treatment, electromagnetic radiation, high-pressure processes, or ultraviolet light exposure [116]. From a technological perspective, postbiotics provide significant advantages over live probiotics, particularly in terms of stability and safety [117]. Postbiotics reduce the risk of infections or adverse immune reactions, making them particularly beneficial for individuals with weakened immune systems or compromised skin barriers [117]. The production and bioactive properties of postbiotics are shown in Figure 3.

This makes them particularly relevant for use in topical formulations for individuals with sensitive or diseased skin.

There are various examples of preclinical and clinical evidence implicating the therapeutic potential of postbiotics, as shown in Table 1.

Postbiotics have been incorporated into a variety of skincare products, including moisturizers, serums, and cleansers, aimed at improving skin health by restoring microbial balance and enhancing skin resilience [128,129,130]. The use of postbiotics in dermatology is supported by a growing body of evidence from both preclinical and clinical studies, which have demonstrated their potential in treating a range of skin conditions from acne to eczema. For instance, lipoteichoic acid (LTA), a key component of the cell wall in Gram-positive bacteria, has shown inhibitory effects on melanogenesis [118]. LTA derived from *Lactobacillus plantarum* suppressed melanogenesis in B16-F10 cells by decreasing cellular tyrosinase activity and downregulating the expression of tyrosinase in a dose-dependent manner [118]. In clinical trials, Majeed et al. demonstrated that LactoSporin, a postbiotic derived from *Bacillus coagulans*, significantly decreased oiliness, pimples, and redness associated with acne, marking it as a candidate for acne treatment [119]. The potential efficacy of postbiotics in reducing acne-related issues could be attributed to their ability to inhibit the enzyme 5-alpha-reductase, as demonstrated in in vitro studies [119]. This enzyme is crucial in the production of hormones that stimulate sebaceous gland activity, creating an environment conducive to the growth of *Cutibacterium acne*, the bacterium often linked to acne development [131]. By inhibiting 5-alpha reductase, postbiotics may help manage sebum production, thus reducing one of the primary factors that support acne formation. Further, Chung et al. developed a multi-strain postbiotic complex (PC) combining *Lactobacillus helveticus* HY7801 and *Lactococcus lactis* HY449 [120]. This complex exhibited antibacterial effects against *Staphylococcus aureus* and *Cutibacterium acnes*, while also modulating inflammatory cytokines and hyaluronic acid, which contribute to the anti-inflammatory response in skin cells [120].

Further, torii et al. demonstrated that oral administration of L-92 derived from *Lactobacillus acidophilus* significantly improved atopic dermatitis in Japanese children by regulating the T helper type-1/T helper type-2 (Th1/Th2) immune axis [121]. Similarly, Lima et al. demonstrated the efficacy of orally administered *Lactobacillus*-derived postbiotics in atopic dermatitis [132]. Moreover, Kim et al. investigated the synergistic effects of combining lactic acid bacteria postbiotics with *Smilax china* L. extract through a cofermentation process, focusing on their potential therapeutic application for atopic dermatitis [133]. This study introduced MB-2006, a fermented product developed by cofermenting *Smilax china* L. extract with *Lactobacillus acidophilus* (LAC) and *Lactobacillus rhamnosus* (LRH), and compared its efficacy to that of the heat-killed probiotics LAC and LRH alone [133]. Their findings demonstrated that MB-2006 was significantly more effective than LAC or LRH alone in reducing pro-inflammatory markers, such as IL-4 and thymic stromal lymphopoietin (TSLP), and in inhibiting the NF-κB pathway and the activation of atopic dermatitis-like HaCaT keratinocyte cells [133]. Furthermore, *Lactobacillus*-derived postbiotics have demonstrated benefits in contact dermatitis models, where they reduced pro-inflammatory cytokines and enhanced skin barrier integrity. *Lactobacillus rhamnosus GG*-derived postbiotics, for instance, reduced inflammation in allergic dermatitis models by modulating immune pathways and strengthening skin barriers [134].

A recent study demonstrated the anti-inflammatory potential of *Malassezia*-derived lipid metabolites such as poly unsaturated fatty acid (PUFA). Malassezin, a natural indole derivative, is produced by the skin-resident fungus *Malassezia furfur* and has been linked with the skin depigmentation disorder pityriasis versicolor [122]. Research indicates that topical application of isolated Malassezin can reduce epidermal discoloration, such as hyperpigmentation caused by photoaging [123], likely due to its selective apoptotic effects on primary human melanocytes. As a unique agent derived from the skin microflora, Malassezin is now available as a commercial ingredient for skin-whitening products, targeting hyperpigmented areas while preserving the pigmentation of normal skin [123].

Likewise, the opportunistic skin pathogen *Cutibacterium acne* produces propionic acid, which holds promise as a safe and effective alternative for treating hyperpigmentation [124]. Another compound, pityriacitrin, an indole alkaloid derived from *Malassezia furfur*, has demonstrated strong UV-protective properties [125]. The UV-absorbing properties of pityriacitrin impact various bacterial and fungal members of the human skin microbiota and show potential for use as postbiotic ingredients in commercial sun protection products [126]. Based on the above findings, it can be postulated that postbiotics, which have the ability to reduce hyperpigmentation and inflammation, could represent a promising approach to treating vitiligo; however, further research is required in order to fully understand the microbiome’s role in vitiligo and to develop effective microbiome-based therapies.

Further, microbiome-targeted therapies are being explored as potential treatments for AA. By increasing proliferation and cell migration, these therapies aim to regulate hair growth. For example, a double-blind, randomized study involving 160 participants was conducted to assess the effectiveness of a gel formulation containing postbiotics for the topical treatment of AA. Among the patients treated, approximately 47.5% experienced complete hair regrowth, 13.75% showed partial regrowth, and 6.25% reported no response [111]. Key mediators of the epithelial cell proliferation and migration of human keratinocytes also increased the expression transforming growth factor beta-1 (TGF-β1), vascular endothelial growth factor A (VEGF-A), fibroblast growth factor 7 (FGF7), and IL-8 genes [135]. In addition, both compounds modulate the expression of filaggrin, involucrin, β-defensin 2, and TNF-α genes [136]. Based on this evidence, it can be speculated that postbiotics could provide a new avenue for treatment strategies.

Recent advancements in microbiome research have opened the door to a variety of interventions that modulate skin microbiomes for improved health and cosmetic outcomes. Among these, postbiotics, prebiotic, and other microbiome modulators have gained considerable attention for their ability to promote skin barrier integrity, reduce inflammation, and treat skin diseases. These microbiome-based therapies aim to restore or maintain the natural balance of the skin’s microbial communities, which is crucial for overall skin health. In the following section, we emphasized the role of postbiotics, prebiotics, and other modulators in skin health, along with their applications in skincare products and treatments.

**Inactivated bacteria (postbiotics) and microbial metabolites as postbiotics in skin health:** Inactivated probiotics, also known as non-viable probiotics, are bacterial strains that have been inactivated or killed but still retain their beneficial properties. A study by Dimarzio et al. demonstrated the antiaging effect of peptides derived from *Streptococcus thermophilus S244* [137]. The mechanism involves an increase in the ceramide level and hydration of forearm. Further, heat-killed *Lactiplantibacillus* *plantarum* have been reported to have an antiwrinkle and UV-protective effect. Moreover, it has been shown to reduce the skin melanin level [138]. Therefore, it can be effective for the treatment of hyperpigmentation. Another study by Kim et al. demonstrated the skin barrier-enhancing effect of dermabiotics derived from *Lactiplantibacillus* *plantarum* [127]. Further, CLS02021 (postbiotic), through topical application, increased skin elasticity and reduced both wrinkles and pore size [139]. Inactivated cells of the bacteria belonging to members of the *Lactobacillaceae* family promote anti-inflammatory responses, reduce skin sensitivity, and improve moisture retention when applied topically. Preclinical studies have demonstrated the efficacy of dead bacteria in enhancing skin health. For example, non-viable *Lactobacillus reuteri* has been shown to reduce skin inflammation and promote the healing of wounds [140]. In clinical settings, dead probiotics have been used in the treatment of conditions such as acne. In this context, Bae et al. demonstrated that heat-killed *Pediococcus acidilactici* (LM1013) inhibits biofilm formation by *Cutibacterium acnes* [141]. Another clinical study showed the efficacy of cofermented postbiotics of TYCA06/AP-32/CP-9/collagen with respect to their ability to combat acne vulgaris [142]. Furthermore, rice flour fermented with *Lactobacillus paracasei CBA L74* was found to be effective in the treatment of atopic dermatitis in infants [143]. Although several dead probiotics have been found effective against skin disease, their mechanistic proof is still missing, which provides scope for further extensive research. Microbial metabolites, another class of postbiotics, are compounds produced by live microorganisms during their metabolic activities. For instance, SCFAs such as butyrate and acetate are known for their anti-inflammatory and immune-modulating properties against dermatitis [144]. Additionally, butyrate reduces skin inflammation and dermal IL-33 expression caused by *Staphylococcus aureus* by inhibiting histone deacetylase [145]. Further, Trompette et al. demonstrated that gut-derived short-chain fatty acids modulate skin barrier integrity by promoting keratinocyte metabolism and differentiation, improving epidermal barrier integrity and ultimately limiting early allergen sensitization and disease development [146]. Moreover, a postbiotic derived from *Lactobacillus reuteri* PTCC1655 has been shown to enhance wound healing by modulating the inflammatory phase, increasing collagen and elastin deposition, and promoting angiogenesis [147]. Additionally, gene expression assays revealed that this postbiotic accelerates healing by upregulating both inflammatory mediators (IL-6 and TNF-α) and anti-inflammatory mediators (TGF-β and VEGF) [147]. Furthermore, a study by Golkar et al. evaluated the wound healing efficacy of three topical cold cream formulations containing postbiotics from *Lactobacillus fermentum*, *Lactobacillus reuteri*, and *Bacillus subtilis* species [148]. All of the formulations significantly accelerated wound healing compared to controls, with the *Bacillus subtilis* cream demonstrating superior results [148]. These findings suggest that postbiotic-infused creams hold promise as effective wound healing treatments (see Table 2 and Table 3).

In the context of skin health, microbial metabolites can improve skin hydration, reduce inflammation, and promote wound healing. For example, lactate, a metabolite produced by *Lactobacillus* species, helps maintain skin moisture by enhancing the production of ceramides in the epidermis [151,152]. Similarly, a tryptophan metabolite of the skin microbiota attenuates inflammation in patients with atopic dermatitis through the aryl hydrocarbon receptor [153].

## 8. Conclusions and Future Perspective

This review has explored the essential role of skin microbiomes in maintaining skin health and the pathogenesis of various skin diseases. Our understanding of the skin microbiome has opened new avenues for therapeutic interventions, with postbiotics, dead probiotics, and microbiome metabolites showing great promise in both skincare and dermatology. Postbiotics and dead probiotics have shown potential in inhibiting inflammation and the immune–inflammation axis and treating conditions such as acne, eczema, and wounds. Microbiome metabolites, i.e., SCFAs such as acetate, butyrate, and lactate, also offer promising therapeutic avenues for treating and preventing skin diseases. Despite significant progress, there are several unanswered questions that require more preclinical studies to understand the precise mechanisms through which postbiotics and prebiotics modulate the skin microbiome and promote skin health and wound healing. Future research should focus on elucidating the molecular pathways through which microbiome modulators influence immune responses, skin barrier function, and microbial composition. Understanding these mechanisms will facilitate the development of more targeted and effective treatments. While there is growing evidence from clinical trials supporting the efficacy of microbiome-based therapies, larger, long-term studies are required to validate their safety and effectiveness across diverse populations.

## Figures and Tables

**Table 1:** Preclinical and clinical studies on postbiotics in skin health.

Model/Population	Postbiotic Type	Outcome	References
In vitro study using B16F10 mouse melanoma cells	Lipoteichoic acid (LTA) derived from *Lactiplantibacillusplantarum*	Inhibits melanogenesis and reduces cellular tyrosinase activity; reduces expression of tyrosinase family members	[118]
Open-label, randomized monocentric study in humans with mild to moderate acne	LactoSporin (derived from *Bacillus coagulans*)	Reduces oiliness, acne, and redness around acne	[119]
In vitro study using human keratinocytes (HaCaT cells)	Postbiotic complex (PC) from *Lactobacillus helveticus* HY7801 and *Lactococcus lactis* HY449	Antibacterial effects against *Staphylococcus aureus* and *Cutibacterium acne*; shows anti-inflammatory activity by modulating inflammatory cytokines; increases hyaluronic acid levels in keratinocytes	[120]
Japanese children with atopic dermatitis	L-92 (derived from *Lactobacillus acidophilus*)	Significantly improves atopic dermatitis by regulating the Th1/Th2 immune axis	[121]
Human skin	Malassezin (from *Malassezia* furfur)	Associated with pityriasis versicolor	[122]
Human skin, in vitro	Malassezin	Decreased epidermal discoloration; reduced photoaging-induced hyperpigmentation; apoptotic effects on primary human melanocytes	[123]
Human skin	Propionic acid (from *Cutibacterium acnes*)	Potential non-toxic solution for hyperpigmentation	[124]
Yeast, bacteria, and humans	Pityriacitrin (from *Malassezia* furfur)	Potent UV-protective properties	[125]
Bacterial and fungal skin microflora	Pityriacitrin (yeast)	UV-absorbing effects on skin microflora	[126]
HDB lysate	*Lactiplantibacillus plantarum*	Enhanced skin hydration; reduced transepidermal water loss (TEWL); diminished skin redness on face	[127]

**Table 2:** Preclinical and clinical studies on dead probiotics in skin health.

Microbial Source	Model/Population	Postbiotic Type	Outcomes	References
*Epidermidibacterium Keratini* (EPI-7)	Healthy women	Ferment filtrate	Significantly enhances skin tone and skin microbiome diversity	[141]
*Pediococcus acidilactici* LM1013	Patients diagnosed with acne vulgaris	Heat-treated	Inhibites acne vulgaris	[149]
*Lactobacillus acidophilus* TYCA06*, *Ligilactobacillus salivarius* AP-32, and *Bifidobacterium animalis* subsp. *lactis* CP-9	Patients diagnosed with acne vulgaris	Collagen cofermentation	Ameliorates redness and inflammation	[142]
Peptides from *Streptococcus thermophilus S244*	Aging population	Sonicated	Increases skin hydration and boost ceramide levels in the skin on forearm	[137]
GMNL6 from *Lactiplantibacillus* *plantarum*	Aged population	Heat-killed	Increases skin hydration and reduces wrinkles; improves skin texture, tone, and UV-induced spots; reduces skin redness and melanin levels on face	[138]
*L. casei* AN177, *Lactiplantibacillus plantarum* AN057, *S. thermophilus* AN157	Aged population	Cofermented metabolites CLS02021	Improves skin hydration and elasticity; reduces pore size and wrinkle depth on face	[139]

**Table 3:** Studies on microbial metabolites in skin health.

Population	Metabolites	Outcomes	References
Adults with dry skin	Lactate	Improves skin hydration and reduced dryness and treatment of moderate xerosis	[150]
Hapten-sensitized mice	Butyrate and acetate	Effective against dermatitis	[144]
Atopic dermatitis-like mouse model	Butyrate	Prevents skin inflammation	[145]
Atopic dermatitis-like skin inflammation	SCFAs	Increase skin barrier integrity by promoting keratinocyte and prevent allergic inflammation	[146]
